# A Cervid Vocal Fold Model Suggests Greater Glottal Efficiency in Calling at High Frequencies

**DOI:** 10.1371/journal.pcbi.1000897

**Published:** 2010-08-19

**Authors:** Ingo R. Titze, Tobias Riede

**Affiliations:** 1 National Center for Voice and Speech, University of Utah, Salt Lake City, Utah, United States of America; 2 Department of Communication Sciences and Disorders, The University of Iowa, Iowa City, Iowa, United States of America; 3 Department of Biology, University of Utah, Salt Lake City, Utah, United States of America; Humboldt University, Germany

## Abstract

Male Rocky Mountain elk (*Cervus elaphus nelsoni*) produce loud and high fundamental frequency bugles during the mating season, in contrast to the male European Red Deer (*Cervus elaphus scoticus*) who produces loud and low fundamental frequency roaring calls. A critical step in understanding vocal communication is to relate sound complexity to anatomy and physiology in a causal manner. Experimentation at the sound source, often difficult in vivo in mammals, is simulated here by a finite element model of the larynx and a wave propagation model of the vocal tract, both based on the morphology and biomechanics of the elk. The model can produce a wide range of fundamental frequencies. Low fundamental frequencies require low vocal fold strain, but large lung pressure and large glottal flow if sound intensity level is to exceed 70 dB at 10 m distance. A high-frequency bugle requires both large muscular effort (to strain the vocal ligament) and high lung pressure (to overcome phonation threshold pressure), but at least 10 dB more intensity level can be achieved. Glottal efficiency, the ration of radiated sound power to aerodynamic power at the glottis, is higher in elk, suggesting an advantage of high-pitched signaling. This advantage is based on two aspects; first, the lower airflow required for aerodynamic power and, second, an acoustic radiation advantage at higher frequencies. Both signal types are used by the respective males during the mating season and probably serve as honest signals. The two signal types relate differently to physical qualities of the sender. The low-frequency sound (Red Deer call) relates to overall body size via a strong relationship between acoustic parameters and the size of vocal organs and body size. The high-frequency bugle may signal muscular strength and endurance, via a ‘vocalizing at the edge’ mechanism, for which efficiency is critical.

## Author Summary

More than 5,000 species of mammals share a basic larynx design. Many of them use the larynx to produce an enormous variability of sounds, but only in a handful of species has the physiology of sound production been studied. It is impracticable in most species because observation requires invasive techniques. Furthermore, many mammals do not spontaneously vocalize if they are manipulated or handled. We have constructed a finite element model of vocal fold tissue vibration on the basis of morphological and biomechanical features of the Rocky Mountain elk vocal organs. Operating within reasonable physiological parameter ranges, it allows the investigation of sound production efficiency as well as selective forces. The model can produce sounds with fundamental frequencies ranging between 60 and 1,200 Hz, covering not only some of the natural vocal repertoire of the elk's high-pitched bugle calls but also those of its close relative, the European Red Deer, who produces low-pitched roaring sounds with a similar anatomy. The approach is of broader interest, first because techniques can be adapted to other mammal species using only landmark anatomical and biomechanical features, and second, because simulations can serve as playbacks for perception studies investigating the role of vocalizations in communication.

## Introduction

Contrary to expectation based on body size, some large male mammals use high-pitched vocalization for display. The dichotomy between low frequency and high frequency calls for vocal signaling of male characteristics is rarely so dramatic as in two closely related cervid species: European red deer (*Cervus elaphus scoticus*) and Rocky Mountain elk (*Cervus elaphus nelsoni*). During the mating season, one species is recognizable by a low frequency roar, while the other is well-known for its high frequency bugle [1], [2]. Acoustic signals in the vocal communication of mammals are generally very complex because various selective pressures shape them [3]. The complexity can be related to natural and sexual selection. For example, a signal is considered honest if reliable information about the sender can be extracted, such as body size or physical strength. An animal's body size or physical strength has important implications for its physiology, ecology, fecundity, or its aggressive interactions and mating success [4]. The male red deer mating call was selected for low vocal tract resonance characteristics that provide reliable information about body size due to interconnected size-dependent factors involved in sound production [5]. In contrast, it is difficult to make the case that body size is signaled by the high fundamental frequency whistle-like bugle (around 1000 Hz) of the elk. Elk calls sometimes contain low frequency components, but not consistently. The signature is the bugle. What provoked the evolution of such calls that would generally be associated with much smaller animals? Here we investigate the physiological tradeoffs related with the production of high and low frequency sounds.

We have simulated red deer and elk calls with a finite-element model of oscillating vocal folds positioned within a laryngeal cartilaginous framework, applying intrinsic laryngeal muscle activations [6] and a wave propagation model of the vocal tract [7] with the goal to better understand the physiology of this intriguing system. The larynx finite element model was based on the anatomy and biomechanics of Rocky Mountain elk and red deer larynges [8], [9, and data presented here].

## Methods

### General Model Design

#### Cartilage framework

Vocal folds are located inside a framework of five cartilages, four of which are critical for phonation modeling (the thyroid, cricoid and two arytenoid cartilages). We recorded 18 measurements from laryngeal cartilages from 2 male red deer (farmed in Wisconsin, USA) and 10 male elk (from hunter-harvested elk submitted to the Colorado Division of Wildlife's chronic wasting disease surveillance program during the 2006 hunting season). The ranges for all 18 measurements overlap between elk and red deer (Text S1, Table S1, Figure S1), although the red deer measurements ranged at the lower end of those from elk.

**Figure 1:**
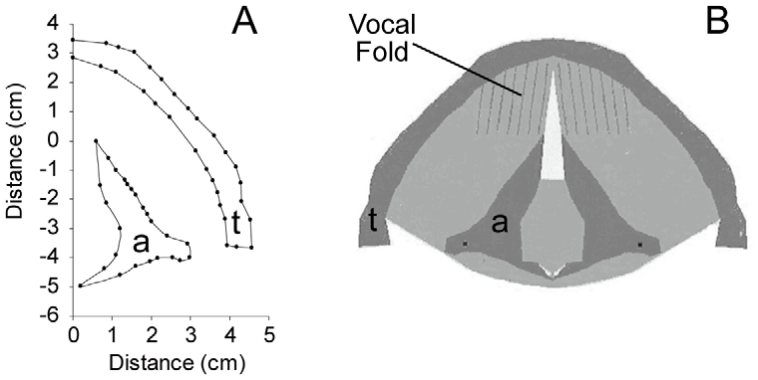
The cartilagenous framework. **A**: Cartilagenous framework measurements of the arytenoid (‘a’) and the thyroid (‘t’) cartilage. **B**: Horizontal section through larynx at the level of the vocal folds as reconstructed virtually from framework measurements.

For the modeling of the cartilage framework we used the larynx of one male 4 year old elk also retrieved from hunter-harvested elk submitted to the Colorado Division of Wildlife's chronic wasting disease surveillance program during the 2006 hunting season. A digitizer (Micro Scribe-3DX, Immersion Corporation, San Jose, CA, USA) was used to measure coordinates of the outlines of cricoid, thyroid, and arytenoid cartilages. The outline of the thyroid and one arytenoid cartilage is shown in Figure 1A. A horizontal cross section at the level of the vocal folds, virtually reconstructed, is shown in Figure 1B. This provides the dorso-ventral dimensions of the vocal fold within the laryngeal framework. The portion of the vocal folds in Figure 1B containing striations (vertical lines) is the vibrating portion that was modeled with a finite-element approach. The striations indicate the fibrous component of the tissue.

#### General approach to finite element model of vibrating vocal fold tissue

Finite element (FE) modeling of vocal fold tissue has seen significant advances in recent years. Alipour et al. [10] introduced the model upon which our current model is built. The innovative approach taken in the current simulation is the combination of physical plant modeling (tissue and air movement) with physiologic modeling that progresses first from muscle activation to muscle mechanics, then to cartilage and soft tissue posturing, then to self-sustained oscillation of tissue, then to glottal airflow, and finally to wave propagation in the vocal tract. We do not claim that all of these components contain equal validity and accuracy. Fluid-structure modeling can usually be subjected to greater validity tests than physiological and biomechanical modeling based on muscle activation. Hence, we have attempted to strike a balance in moving these components forward in proportionate steps, realizing that the complete simulation is never better than its weakest link.

#### Vocal fold tissue simulation

**Figure 2:**
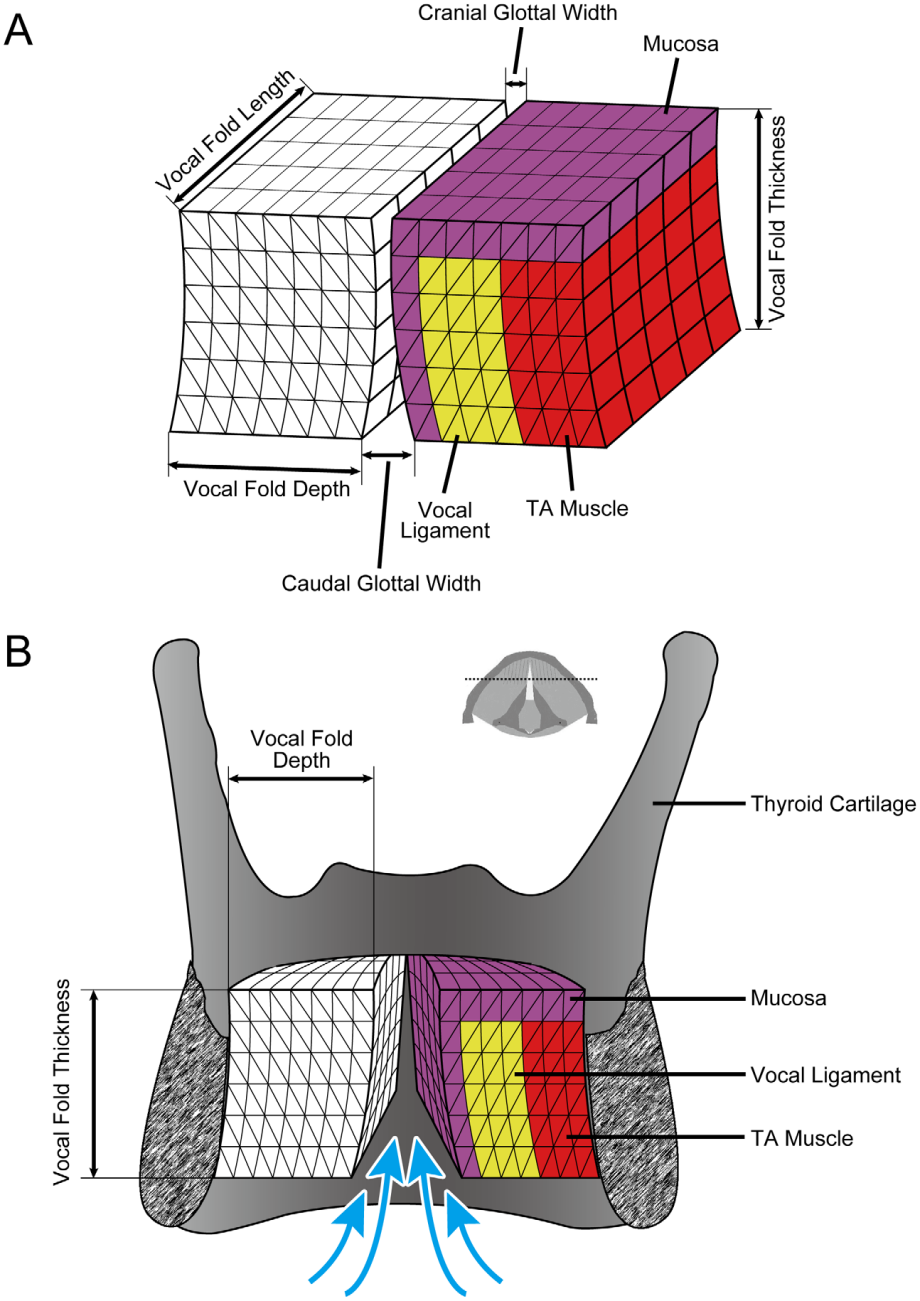
3-D FE model of the vocal folds. **A**: Isolated vocal folds. **B**: Frontal section through the thyroid cartilage and the vibrating portion of the vocal folds shown in Figure 1, perpendicular to the fibers. The triangular element mesh was 12×14 elements for each of 5 layers along the fibers, or along the vocal fold length. Mucosa, ligament, and TA muscle are shown in color for the left vocal fold. The small inset about the larynx is the same top view of the vocal folds as in figure 1 indicating the cross section level with a dotted line.

The fundamental frequency (F_0_) is the lowest of a spectrum of frequencies in the airflow from the lungs that is modulated by vocal fold oscillation. Simulations of vocal fold oscillations are sensitive to viscoelastic and geometric parameters [e.g. 11], [12], [13]. The soft tissue of the vibrating portion of each vocal fold was divided into triangular elements in the coronal plane and into rectangular layers in the ventro-dorsal direction (along the length of the vocal folds) as shown in Figure 2. The number of elements was chosen to capture two principal modes of vibration [14], [15]. These modes are based on a half-wavelength standing waves in the dorso-ventral direction and a half-wavelength standing waves in the caudo-cranial direction on the vocal fold surfaces. We used 12 elements in the caudo-cranial direction, which would be 24 elements per wavelength, satisfying the Courant criterion. In the dorso-ventral direction, the number of layers was restricted to 5. The contiguous open sections along the length of the glottis are combined into a single flow channel, in which a modified Bernoulli flow calculation is used that includes a correction for flow separation from the channel walls [14]. The restriction of five layers in the dorso-ventral direction avoids excitation of a mode in which the dorsal and ventral part of vocal fold move out of phase [14] which would require a two-dimensional glottal airflow calculation, that is currently not implemented.

Within each element, the displacement vector was two-dimensional (vibrational deformation occurring only in the coronal plane), but different for each layer in the ventro-dorsal direction, i.e. along the length of the vocal folds. The material was considered transversally isotropic, meaning that it was isotropic in a plane transverse to the dorso-ventral tissue fiber direction. In essence, the tissue was a fiber-gel compound. Tissue fibers connected the rectangular layers along the dorso-ventral length of the vocal folds. The viscoelasticity of the fibers has been measured for cervids [8], [9, Riede, unpublished data]. The viscoelasticity of the gel in the transverse (coronal) plane has not been measured for cervids, but was assumed to be similar to that of humans [10]. Tissue was considered to be nearly incompressible at sonic frequencies, with a planar Poisson ratio of 0.9 (1.0 being completely incompressible in a plane, which cannot be used because of computational instability). The constitutive equation for the gel substance was that of linear viscoelasticity, defined by the shear elastic modulus, the Poisson ratio, and the shear viscosity [16]. The constitutive equation for the fibers was that of one-dimensional nonlinear viscoelasticity, following a traditional Kelvin approach for soft tissue [8], [17]. Muscle fibers had active contractile properties.

The temporal integration step size (44.1 kHz) was chosen to match the requirements for wave propagation in the vocal tract (to be described later). The source calculations at twice or half this sampling frequency had no effect on the tissue or airflow, except at frequencies above 900 Hz. The sampling frequency was increased by a factor of 50 (2.2 MHz) to reach stability and convergence at the highest frequencies of interest.

Vocal fold collision has been modeled previously with finite element techniques [e.g. 18], [ 19]. It was included in the current model. When a nodal point overlapped slightly with one on the opposite vocal fold, an exponential soft return to no-overlap over several time steps was implemented to avoid a sudden jerk on the tissue.

A summary of the mathematical construct for vibration is given in Alipour et al. [10] and a fully detailed description of the underlying vocal fold biomechanics is given by Titze [11]. The material properties for the current simulation are listed in Table 1.

**Table 1:** Input parameters used in model.

Measured Parameter	Value
Vocal fold static length L	2.6 cm
Vocal fold depth, D	1.68 cm
Vocal fold thickness, T	1.8 cm
Rostral glottal width	0.06 cm
Caudal glottal width	0.02 cm
Range of lung pressure *PL*	0.5–8 kPa
Longitudinal Poisson's ratio for all tissue layers	0.1
Transverse Poisson's ratio for all tissue layers	0.9
Longitudinal shear modulus of the body	variable with length
Longitudinal shear modulus of the cover	variable with length
Longitudinal shear modulus of the ligament	variable with length
Transverse shear modulus of the body	0.5 kPa
Transverse shear modulus of the cover	0.5 kPa
Transverse shear modulus of the ligament	0.5 kPa
Muscle viscosity	2 poise
Mucosa viscosity	2 poise
Ligament viscosity	2 poise

#### Nonuniform material characteristics

Mammalian vocal folds consist of several layers of tissue in the coronal plane (Figure 2) [20]. Along the surface is stratified squamous epithelium. Underneath is the *lamina propria*. The *lamina propria* consist of extracellular matrices of collagen and elastin proteins as well as glycosaminoglycans like hyaluronan, and a few cells (mostly fibroblasts and some macrophages). In humans and some nonhuman mammals, the *lamina propria* are subdivided into superficial, intermediate and deep layers [8], [9], [21], [22]. Lateral to the *lamina propria* is a muscle (*musculus thyroarytenoideus*, henceforth labeled TA muscle) which demonstrates passive and active stress response characteristics.

**Figure 3:**
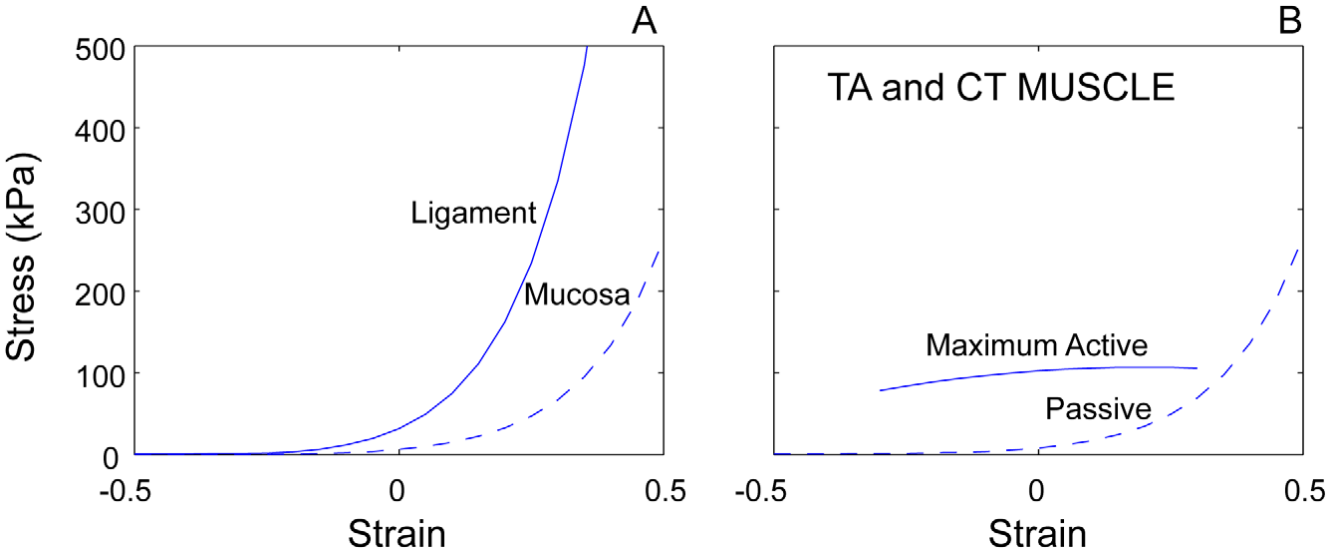
Stress-strain curves for vocal fold tissue. **A**: Stress-strain curves vocal ligament and mucosa. **B**: active and passive muscle tissue (TA, thyroarytenoid muscle; CT, cricothyroid muscle).

- Passive components: Passive viscoelastic properties of elk vocal fold mucosa and ligament have been measured along the dorso-ventral axis (Figure 3 A) [8].
- Active components: Approximated maximum active and passive stress for the thyroarytenoid and cricothyroid muscle tissues are based on *in vitro* measurements in dogs [6] (Figure 3 B). For simulation, activation of the TA and CT muscles (hereafter symbolically referred to as: a_TA_ and a_CT_, respectively) are expressed as a percentage of the maximum active stress. The maximum active curve is centered around the cadaveric vocal fold resting length. For high strains above about 0.2, the ligament stress dominates all other stresses.

Vocal fold strain was calculated on the basis of an empirical relation obtained on anesthetized domestic dogs [23]:
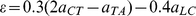
(1)where a_LC_ is activation of the lateral cricoarytenoid muscle, which was held constant at 0.45 in all our simulations to maintain uniform vocal fold adduction.

Modeling mucosa, ligament and muscle layer with finite element methods has advantages over low-dimensional lumped-element approaches because boundaries between the layers can be clearly differentiated [e.g. 24]. Effective mass and stiffness in vibration are then self-regulated. But there are still limitations. The epithelium is too thin (on the order of 0.05 mm) to be modeled as a separate layer. Hence, it is usually combined with the superficial layer of the *lamina propria* and referred to as *mucosa*. Likewise, the intermediate and deep layers of the *lamina propria* are combined and identified as *ligament*. For the currently described tissue construct, then, we have a mucosa, a ligament, and a muscle, as shown in the right half of Figure 3. The left half has the same properties.

#### Boundary conditions

There are six boundaries for the vibrating portion of each vocal fold. Tissue vibration is constrained to be zero on surfaces laterally, ventrally, and dorsally. This corresponds to surfaces at or near the thyroid cartilage and the arytenoid cartilage (Figure 2), where tissue fibers either originate or insert. Tissue vibration is unconstrained cranially, caudally, and medially. This corresponds to the cranial, caudal and medial surfaces of the vocal folds, where aerodynamic and acoustic pressures are applied (Figure 2). The boundary conditions are formulated in terms of forces or displacements at the nodes of each finite element. As typical in finite element methods, interpolation functions (in integral form) are derived to express displacements and velocities inside each element.

#### Aerodynamic forces

A modified Bernoulli approach was used to express the pressures on all three free surfaces. The modification to Bernoulli's energy equation included three aspects. First flow separation in the glottis (to form a jet) was assumed to occur when a downstream area was greater than 1.2 times the minimum glottal area [25], or at glottal exit. Second, the overall glottal area was a summation over five ventro-dorsal sections. Third, acoustic waves propagating in supraglottal and subglottal airways were superimposed upon steady (Bernoulli) pressures at glottal entry and exit. The details of these modifications are found in [26], [27].

Subglottic pressure data during phonation are available for a few mammals (in vivo measurements: human: 0.3–6 kPa, [28]; bat: 0.5–7 kPa, [29]; horses: 0.5–8 kPa [30]; excised larynx experiments in various species: 0.3–5 kPa, [31], [32], [33], [34]). Maximum expiratory tracheal pressure has been measured as 14 kPa in human [35]. We therefore consider a range between 0.2 and 12 kPa realistic when small to large effort is exerted.

#### Postural forces

Vocal fold dynamics includes *a*) relatively slow and large movements affecting adduction and elongation of the vocal folds, and *b*) fast and small amplitude oscillatory movements in which various layers of the vocal fold are vibrating [36], [37]. Vocal fold posturing with realistic biomechanics and muscle activation represents the single most important advance of the current model from other vocal fold models. The intrinsic laryngeal muscles (cricothyroid muscle, CT; thyroarytenoid muscle, TA; interarytenoid muscle, IA; lateral crico-arytenoid muscle, LCA, posterior cricothyroid muscle, PCA) are modeled with elastic, viscous and contractile elements. Parameters for the biomechanics of postural movements were the activities of the above muscles, symbolized by a_CT_, a_AT_, a_LC_, a_IA_, and a_PC_. Each of these activities could range from 0.0–1.0 (0–100%). Large amplitude movements involve the arytenoid cartilage rocking on the cricoarytenoid joint. The IA and LCA muscles mostly regulate this movement. Three-dimensional adduction has been implemented as a rocking–sliding motion [38], but a two-dimensional equivalent is used here, resulting in effective joint rotation and translation [39]. The elk (our own investigation) and red deer [40, our own investigation] possesses the same set of muscles with similar fiber orientation, and therefore a similar set of posturing forces was applied.

#### Vocal tract design

**Figure 4:**
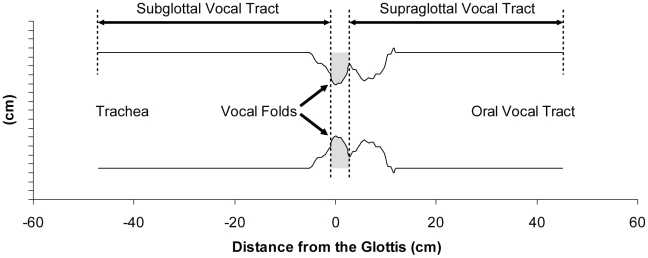
Vocal tract simulation. The cross sectional areas of the vocal tract are indicated relative to the location of the glottis (space between vocal folds). The vocal folds inside the dotted square were made of an element mesh as indicated in Figure 2.

The air way below and above the larynx is considered the vocal tract. This column of air has resonant modes that selectively allow certain frequencies to pass and radiate from the mouth (or nostrils) better than others. These resonances of the vocal tract, along with the spectral peaks they produce in the radiated signal, are called formants. Vocal tract resonances are highly dependent on the geometry of the vocal tract [e.g. 41], [42]. In this first approach we focused on source acoustics and therefore kept vocal tract dynamics simple. Vocal tract length was estimated from measurements in 10 adult male elk. Cadavers were retrieved from hunter-harvested elk submitted to the Colorado Division of Wildlife's chronic wasting disease surveillance program during the 2006 hunting season. The length of supraglottal oro-pharyngeal vocal tract cavity was measured with a string which was position intra-orally from the tip of the lower incisive to the cranial edge of the thyroid cartilage (N = 10, 47.3±3.6 cm). The supraglottal tract was modeled with 112 serial tubelets of equal length, for a total length of 44.5 cm (Figure 4). The length of the subglottal vocal tract (a short intra-laryngeal distance and the trachea) was measured from the upper edge of the cricoid cartilage to the bifurcation in a relaxed trachea (N = 10, 44.2±1.9 cm). The subglottal tract was modeled with 120 serial tubelets, each 0.397 cm in length, for a total length of 47.6 cm (Figure 4). In order to determine cross sectional areas along the trachea's length, the tracheal tube was sectioned into several rings and their cross sections were photographed in one 4-year old male elk. The area of each ring was measured against a reference of known length with *Image* software (developed at the Research Services Branch of the National Institute of Mental Health, NIMH). The cross sectional area of this part of the vocal tract was estimated at 13 cm^2^. The relatively rigid intralaryngeal part of the vocal tract (sub- and supraglottal) was determined by injecting dental cast. The cast was sectioned into 4mm thick slices. Each slice was photographed and its area was measured against a reference of known length with NIH image software. Unfortunately, the cadaveric vocal tract airway shape can only be regarded as an approximation to the shape when the animal is vocalizing.

A wave reflection algorithm was used to calculate incident and reflected pressure waves above and below the glottis, which were then included in the driving forces on vocal fold tissue [14], [24], [43]. It is important to note that the vocal tract configuration was kept constant in all simulations presented here.

Radiated power output from the mouth (*P*_rad_) was computed from knowledge of the radiation impedance for a given mouth opening [44], which dictated the reflection and radiation of acoustic waves at the mouth end. Aerodynamic power (*P*_air_) was computed as:
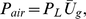
(2)where P_L_ is the lung pressure and U_g_ is the mean glottal flow.

Vocal tract resonances were measured by an impulse response. A glottal flow pulse of one sample duration and 10 l/s magnitude was introduced at the input of the vocal tract, which caused multiple wave reflections that dissipated over time. A Fourier analysis of the decay response yielded the resonance structure.

### Muscle Activation Plot

The working range of the model is summarized using key physiological features in a muscle activation plot (MAP) in which CT activity is plotted against TA activity. Furthermore, the plot indicates the relationship between muscle activity, vocal fold strain, subglottic pressure and F_0_. The principal goal here was the identification of ranges of identical F_0_ ranges in so-called iso- F_0_ lines. These lines indicate points at which stable phonation near phonation threshold pressure (PTP) can be maintained. PTP is the minimum subglottal pressure required to initiate vocal fold oscillation [45]. In order to identify such points, repeated simulations were necessary. The protocol was as follows: For a given TA activity, CT activity and subglottic pressure were increased stepwise until a stable phonation at the goal fundamental frequency was achieved. Each simulation resulted in a wav-file which was visually inspected. Every sample of phonation was examined to determine periodicity and to calculate F_0_ by zero crossing or peak picking methods. If the oscillation was neither growing not damped, but survived for at least 10 cycles near threshold because glottal flow was nearly sinusoidal, it was considered stable and counted toward one of the 175 simulations (Figure S2). Each simulation was about 200 ms in duration. Based on these 175 simulations, iso-fundamental frequency lines were created that indicate a range of CT and TA activity combinations for which an identical F_0_ can be achieved with roughly the same phonation threshold pressured.

### Glottal Efficiency Calculation

Glottal efficiency (E_g_) was computed as described in the literature (e.g. [28]):
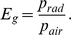
(3)Finally, the sound intensity level (SIL) at a 10 m distance was computed from the radiated output power as:
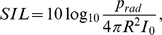
(4)where R = 10m and I_0_ is the ISO standard reference intensity, 10^−12^ W/m^2^.

### Sound Analysis

Simulated elk and red deer calls were sampled at 44.1 kHz, typically of 2 s duration. Some signals were simulated with a 1 s ramp-up in muscle activity and muscle activity was held constant during the second half of the call (“elk calls”), signals were simulated with constant muscle activities throughout the 2 s duration (“red deer calls”). All spectrographic measurements were made in the second half of a call.

Natural vocalization for comparative purposes were collected from elk and red deer. Male elk were recorded between September and November of 2006 and 2007 in the Rocky Mountain National Park, Colorado, USA, with a Marantz PMD 222 tape recorder and Sennheiser microphone (ME80 head with K3U power module; on ChromeSuper tapes 60 min). Red deer calls were recorded in the Müritz National Park in northern Germany in September 1999 (same recording equipment as in Colorado). Vocalizations were sampled at 44.1 kHz. All signals were analysed based on narrowband spectrograms and measurements therein using PRAAT [46].

## Results

### Fundamental Frequency Range

**Figure 5:**
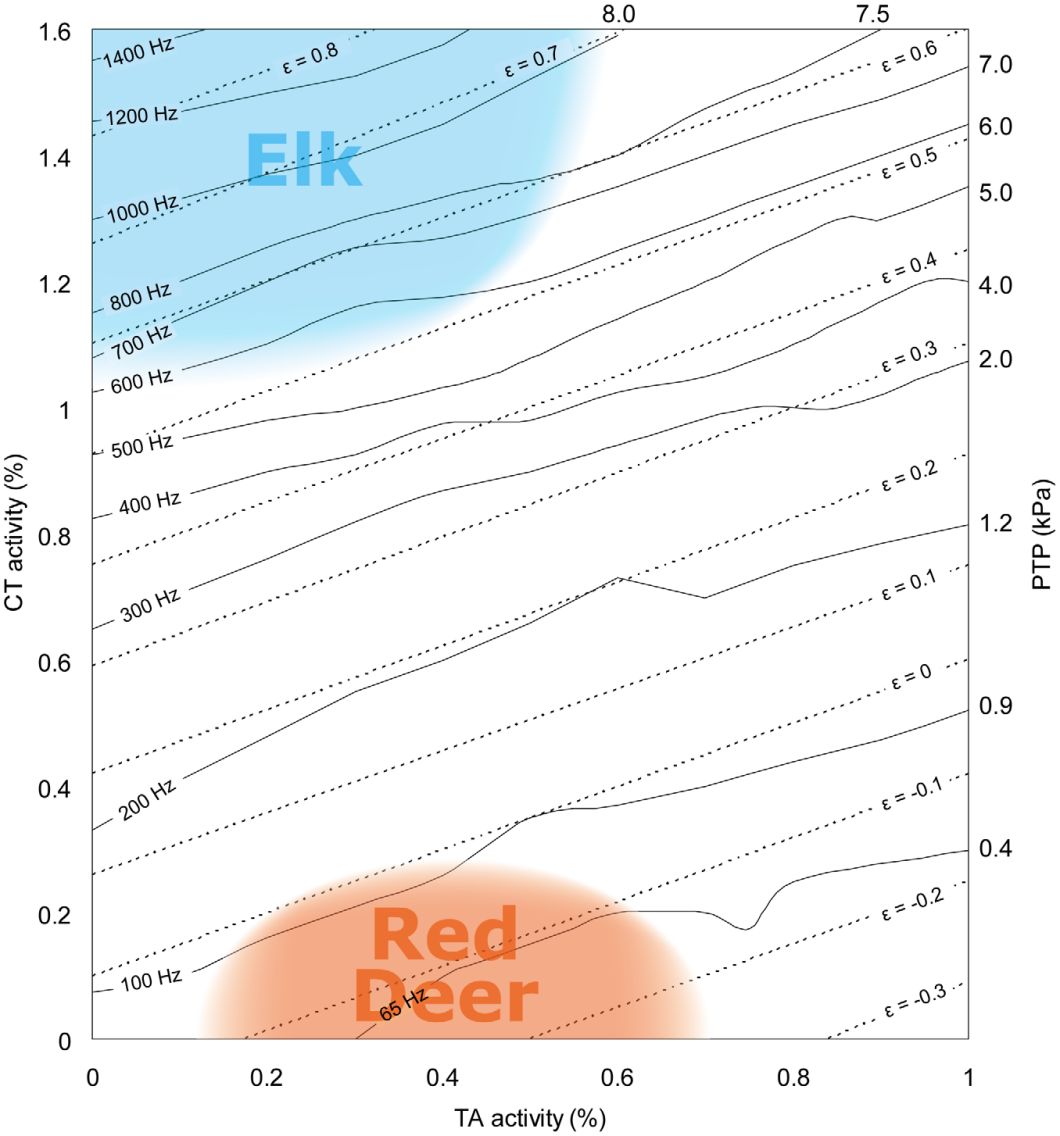
Muscle activation plot (MAP). Iso-fundamental frequency contours for self-sustained vocal fold oscillation (solid lines, frequencies in Hertz, Hz) based on 175 simulations (Figure S2). They indicate where vocal fold oscillation can be maintained at a constant fundamental frequency near phonation threshold pressure, which is indicated in kPa on the right and top axis of the MAP. Iso-strain curves (dashed lines, strain ε) indicate the elongation of the vocal fold in order to achieve a certain tension of the oscillating tissue.

A large fundamental frequency (F_0_) range (60 Hz–1400 Hz) for self-sustained vocal fold oscillation was obtained when cricothyroid (CT) activity and thyroarytenoid (TA) activity were varied. In Figure 5 iso-fundamental frequency lines (solid lines) are plotted along with iso-strain lines (dashed lines). Each PTP value shown along the right margin of the MAP corresponds to an iso-F_0_ line. Note that PTP are approximations and vary slightly over each curve (not more than 5%). Elk vocalizations are near the top of the muscle activation plot and red deer vocalizations are near the bottom.

Vocal fold oscillation could be self-sustained at a minimum F_0_ of 65 Hz (subglottic pressure = 0.4 kPa; a_TA_ ranging from about 0.3–1.0 and a_CT_ activity ranging from 0–0.3), and at a highest F_0_ of 1400 Hz (subglottic pressure = 12 kPa; a_TA_ less than 0.05, a_CT_ activity approx. 1.6). Because the simulation produced iso-F_0_ *lines* rather than single coordinate F_0_ points, any F_0_ could be produced over a range of muscle activity combinations.

A value of a_CT_ activity above 1.0 poses no physiological non-reality. It simply means that the values are larger than what would be considered about maximum for average domestic dog muscle contraction, which was used as a normalization factor. Muscle activation greater than 1.0 could also mean that the muscle is assisted by laryngeal strap muscles to increase vocal fold length, a well-known phenomenon in human high-note singing. Note that for F_0_ as high as 1200 Hz, the vocal fold strain requirement is about 0.8, or 80% elongation of the vocal folds.

F_0_ can be predicted by the formula for a vibrating string, assuming that the ligament is the string,
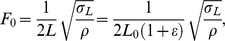
(5)where *L*_0_ is the resting length (2.6 cm from Table 1), *ε* is the strain from Figure 5 (dashed lines) or Equation 1, *σ*_L_ is the ligament stress from Figure 3, and *ρ* is the tissue density (1.04 g/cm^3^). As an example, for *ε* = 0.3 the stress *σ*_L_ = 400 kPa (4×10^6^ dyn/cm^2^), producing an F_0_ of 296 Hz. Note that the 300 Hz iso-F_0_ line in Figure 5 is close to the *ε* = 0.3 line, but the iso-F_0_ line is not straight, for reasons explained in a section below (Source-vocal tract interaction and irregular vocal fold oscillation).

### Simulated Elk and Red Deer Vocalizations

**Figure 6:**
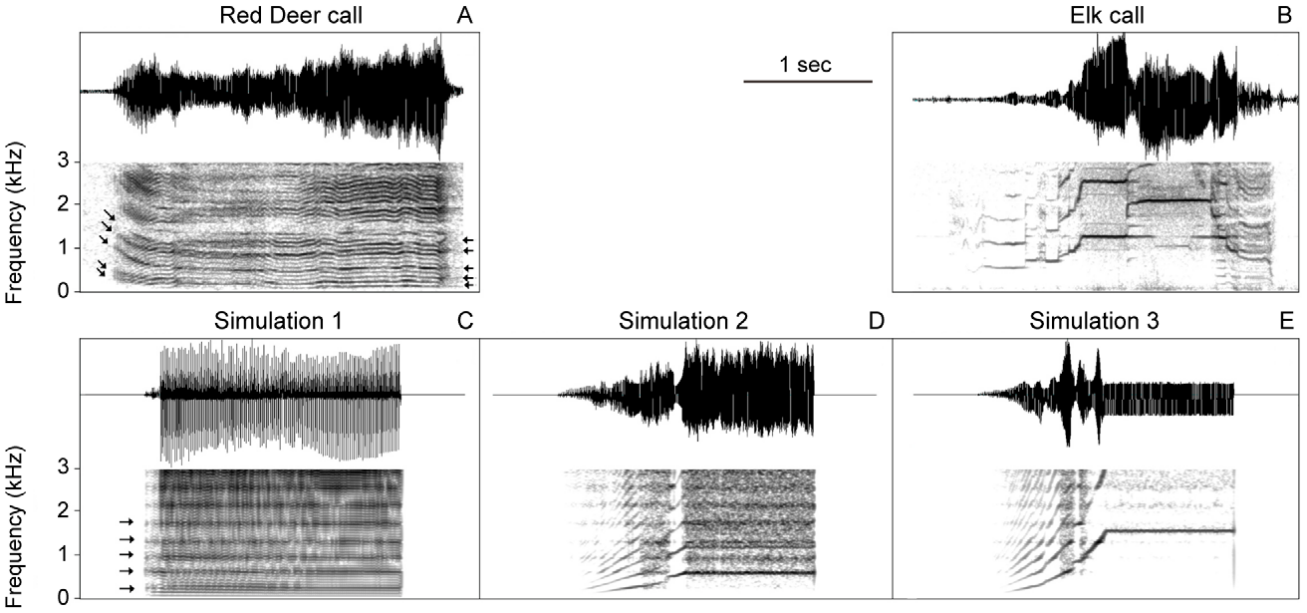
Natural and simulated calls. Oscillograms (upper panels) and spectrograms (lower panels) of a red deer call (**A**), an elk call (**B**) and three sound simulations (**C**, **D**, **E**). Fundamental frequency of simulations is 60 Hz (in **C**), 900 Hz (in **D**) and 1500 Hz (in **E**). All simulations last 2 seconds. Note that intrinsic noise of the nonlinear system indicates the presence of resonance frequencies even if F0 is above those resonances. Arrows indicate resonance frequencies, which are identical in all three simulations, but they are best visible when the source signal has a low fundamental frequency.

A natural red deer call, shown in Figure 6A, is characterized by a F_0_ of around 100 Hz. A rich harmonic spectrum allows formants to be seen. The elk call of approximately the same duration shows a F_0_ that starts at 550 Hz and successively increases to a maximum of 2100 Hz (Figure 6B). The increase occurs partly in a smooth upward glide and partly in frequency jumps. An interesting characteristic is the occurrence of noise between the harmonics. This noise sometimes highlights the formant characteristics, which would normally not be visible with only the harmonics present. This noise presumably arises from air turbulence at glottal exit and complex vibrational modes [11] of the vocal folds.

The model is able to simulate most of the above features. A sound with low F_0_ (65 Hz) and rich harmonic spectrum and clearly visible formant structures is shown in Figure 6C. At different points in the MAP, call components like an elk bugle can be produced, also showing smooth upward gliding F_0_ (Figure 6D, E) and nonlinear phenomena such as sudden frequency jumps (Figure 6E) or chaotic call segments with harmonic windows (Figure 6D). Furthermore, the noisy components between the harmonics are also clearly visible and perceivable in the model sound (Figure 6D, E). Berry et al. [47] have shown that chaotic vibration can be produced with only two or three of the lowest modes of vibration (the empirical eigenmodes related to the cranio-caudal and latero-lateral modes described earlier) if these modes are desynchronized. The noise in our model does not stem from random number generators, but rather from this mode desynchronization when driving forces are large.

### Power Output

For steady phonation, physiological input variables to the simulation model were lung pressure, muscle activations a_CT_, a_LC_, and a_TA_, and simulation time. All other parameters were held constant.

Male elk bugles as well as red deer roars are powerful displays. For example, sound amplitudes in elk calls can reach 90 dB at a 5 m distance (measured in a farmed elk, our own unpublished data), which would correspond to 84 dB at 10 m according to the inverse square law. Calculations from the model are for a 10 m distance from the mouth.

**Figure 7:**
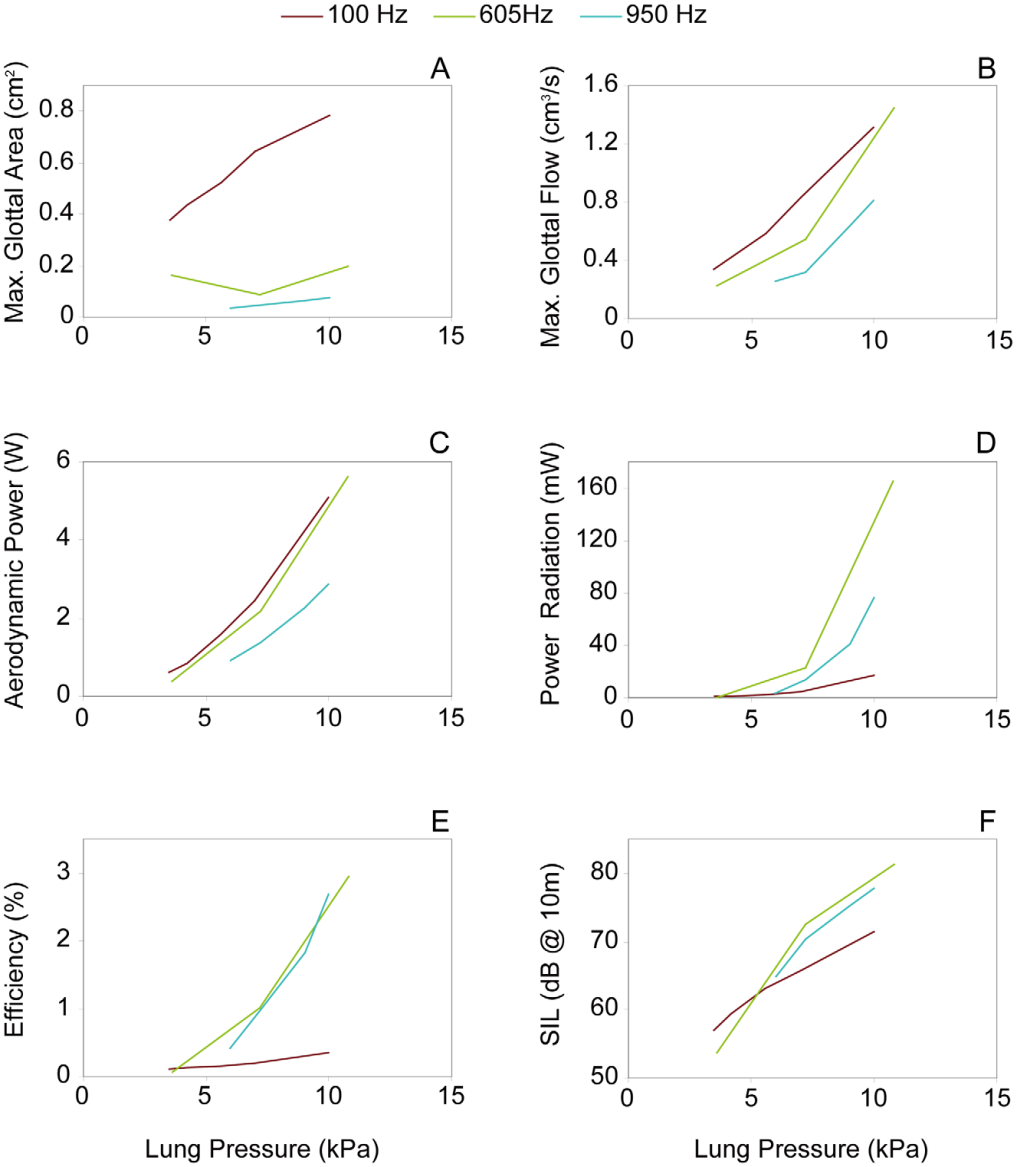
Efficiency measures across the phonation range of an elk/red deer larynx. Six main parameters explaining the energy transfer at the laryngeal sound source and as they change with applied lung pressure. **A**: Peak glottal area, **B**: peak glottal airflow, **C**: aerodynamic glottal power, **D**: radiated sound intensity level. **E**: Radiated Power, **F**: Glottal efficiency.

The following results show six components relevant to laryngeal sound production and their dependence on lung pressure. These are peak glottal area (Figure 7A), peak glottal airflow (Figure 7B), aerodynamic glottal power (Figure 7C), radiated power in mW (Figure 7D), glottal efficiency (Figure 7E), and radiated sound intensity level (at a 10 m distance from the end of the vocal tract tube) (Figure 7F).

The peak glottal area remains small for high F_0_, suggesting that the elk cannot bend the ligament much to allow the glottis to open widely. The glottal area never exceeds 0.2 cm^2^ in the high F_0_ vocalization (Figure 7A). Basically the ligament is a rather inflexible “beam” surrounded by soft, watery tissue. The soft tissue helps to create self-sustained oscillation in the form of a mucosal surface wave [34], but the deeper part of the vocal fold (ligament and muscle) never gain much vibrational amplitude. For low F_0_ and high pressure, however the peak glottal area reaches 0.8 cm^2^.

For high F_0_ and large lung pressure (4 to 10 kPa), large flows are forced through a small glottal opening (up to 1500 cm^3^/s peak flows) (Figure 7B). In general, even larger airflows are maintained at low F_0_ (for example a factor of 2 difference between 100 Hz and 950 Hz at 8 kPa; in Figure 7B). One important implication is that in low F_0_ calls, vital capacity may limit the call duration, given that on the order of 1 liter of air is forced through the glottis in a second.

Aerodynamic power (Figure 7C), which is the product of lung pressure and mean glottal flow, rises to 5 W, which is an order of magnitude higher than in humans for high effort phonation [48]. As F_0_ increases, the reduction in aerodynamic power is linked directly to the lower mean glottal airflow, which in turn is linked to a smaller mean glottal area. Thus, at 950 Hz, there is only 3 W of aerodynamic power at 10 kPa of lung pressure.

Radiated power from the mouth is shown in Figure 7D. An important factor in this is that acoustic radiation from a localized oscillating source quadruples with every doubling of frequency [49]. Thus, going from 100 Hz to 800 Hz should increase radiated power by a factor of 64, all else remaining the same. Figure 7E shows a factor of about 6 from 100 Hz to the higher frequency curves, but smaller airflows and smaller airflow derivatives account for the more moderate increase in F_0_. Glottal efficiency, as defined in Equation 3, is shown in Figure 7E. Note that high F_0_ calls are much more efficient than the 100 Hz call, which is again a function of radiation from the mouth. At 100 Hz and 10 kPa of lung pressure, glottal efficiency is 0.4%, whereas at 605 Hz and 950 Hz it rises above 2%. In either case, most of the aerodynamic power is not radiated from the mouth, but is dissipated as kinetic energy loss at the glottis and in the vocal tract.

Finally, sound intensity level at 605 Hz rises to about 80 dB at an assumed mouth-to-microphone distance of 10 m (Figure 7F). At the lowest F_0_ shown (100 Hz), the radiated sound intensity level is 10 dB lower. This illustrates that the elk may actually have an advantage at high frequency sound productions. High intensity sounds can more easily be produced with large lung pressures at high frequencies. But the cost is great since this requires a large and stiff ligament as will be explained in the next section.

Vibrational amplitude grows with lung pressure, which was raised from phonation threshold pressure to as high as 10 kPa. The amplitude at large lung pressures (>5 kPa) was on the order of 5 mm at F_0_ = 100 Hz, but only on the order of 1 mm at 600 Hz. At F_0_ = 100 Hz, vocal fold oscillation was characterized by large oscillation on the cranial end and caudal edges (Figure 2), but not so large in the center. This characteristic vibration pattern refers to the caudo-cranial mode mentioned earlier [15]. This means also that there was not a lot of contact in the middle of the vocal fold during each cycle, but significant contact between the upper and lower edges. For the high-pitched sounds, the amplitudes were very small, on the order of 0.2 mm at 1200 Hz.

### Source-Vocal Tract Interaction and Irregular Vocal Fold Oscillation

The sound source can be independent or interactive with the vocal tract filter [27]. In a linearly coupled source-filter system the source frequencies are produced independently of the acoustic pressures in the airways. The resonance frequencies of the vocal tract shape the source spectrum, giving rise to formants. The second mechanism is nonlinear coupling, where the acoustic airway pressures contribute to the production of frequencies at the source [27]. Nonlinear coupling has been demonstrated in *in vivo* studies and in modeling approaches [e.g. 42], [50]. In nonlinear coupling, the transglottal pressure includes a strong acoustic component. Weak coupling is obtained when the glottal impedance is high and the vocal tract tube input impedance is low, whereas strong coupling (nonlinear interaction) is obtained when the impedances are comparable.

The curvatures in the iso-F_0_ lines of Figure 5 deserve some attention from the point of view source-vocal tract interaction [27]. When the acoustic vocal tract reactance is inertive, F_0_ is lowered slightly. Conversely, when the acoustic vocal tract inertance is compliant, F_0_ is raised slightly. But harmonics of F_0_ also play a role in this. Suffice it to say, for the purpose of this paper, that F_0_ can fluctuate above and below the natural frequency of the tissue fibers because self-sustained oscillation is (in part) governed by acoustic interaction with the vocal tract.

**Figure 8:**
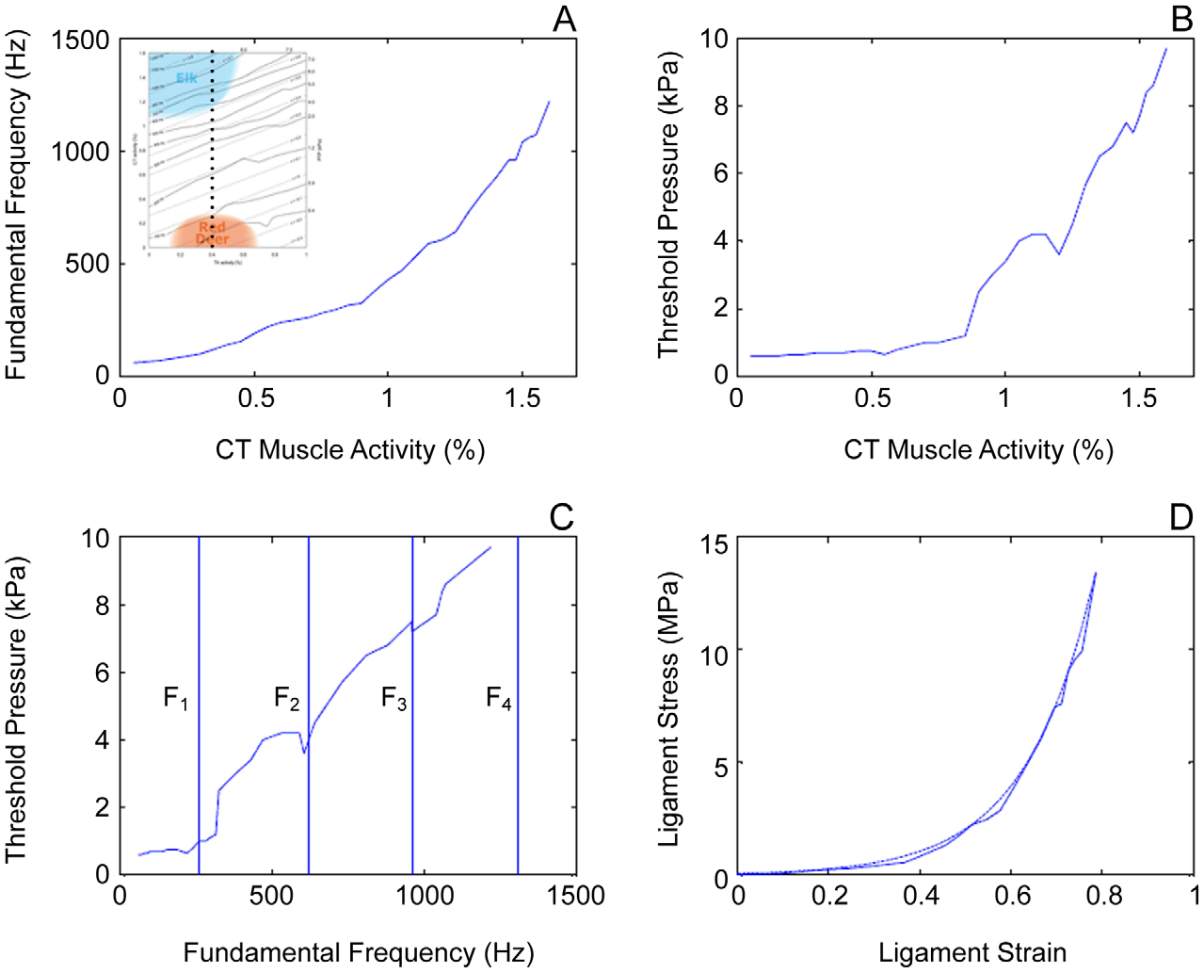
Results of taking a path through the MAP. Taking a path through the MAP of Figure 5 along an imaginary line at a_TA_ = 0.4 (indicated by a dotted line in the inset in **A**), incrementing a_CT_ in steps of 0.05 from bottom to top leads to characteristic changes of acoustic parameters. **A**: fundamental frequency versus a_CT_, **B**: phonation threshold pressure versus a_CT_, **C**: phonation threshold pressure versus fundamental frequency, **D**: ligament stress plotted against strain.

We tested whether vocal tract interaction could have direct effects on the phonation threshold pressure. We found that phonation could be sustained better if F_0_ is near a resonance frequency. Figure 8 shows results of taking a detailed path through the MAP of Figure 5. The path was a vertical line at a_TA_ = 0.4, incrementing a_CT_ in steps of 0.05 from bottom to top. Figure 8A shows F_0_ versus a_CT_. The curve is relatively smooth. Figure 8B is a plot of phonation threshold pressure as a function of a_CT_. Note that phonation threshold pressure generally rises with a_CT_ , but there are some peaks and valleys. To identify where these peaks and valleys occur, phonation threshold pressure is plotted against F_0_ in Figure 8C, with vertical lines showing the formant frequencies F_1_, F_2_, F_3_, and F_4_. The formant frequencies were determined to be 260, 620, 960, and 1370 Hz from an impulse response. Note that phonation threshold pressure is always lower in the vicinity of a formant, which is a clear indication of source-vocal tract interaction. On average, however, phonation threshold pressure is proportional to F_0_. It takes on the order of 9.0 kPa of pressure to initiate phonation at 1200 Hz.

The ligament stress required at such an F_0_ is very large by vocal fold standards. This is shown in Figure 8D. The solid line represents actual finite-element model computations and the dashed line is the simple string model (Equation 5) predicted from the strain ε and the ligament stress σ_L_. For 1000 Hz, the ligament stress is about 10 MPa. This is in order of magnitude equivalent to stress developed in the anterior cruciate ligament (ACL) of the knee in humans [51]. Riede et al. [9] have shown that a stress of this magnitude can rupture the ligament in isolation, indicating that the *in vivo* ligament is probably operated at its mechanical limits. Maintaining a ligament stress of up to 10 MPa for several seconds requires not only large muscular effort, but also a stable laryngeal framework and exceptional material properties to prevent injury. At low F_0_ the mechanical stress due to tensile strain is much smaller, but shear strain due to an increased oscillation amplitude and vocal fold collision may be larger.

## Discussion

Here we have begun the application of FE modeling to a nonhuman larynx, integrating fluid structure interaction with acoustics, morphology, muscle physiology and biomechanics. Just like in other areas of functional morphology [e.g. 52], [53], the virtual sound production system relies on the validity and accuracy of all input parameters (here: viscoelastic properties of soft tissue; active and passive properties of muscles; posturing of laryngeal cartilages; laryngeal airflow; wave propagation in the vocal tract; vocal tract dynamics etc.). The weakest link scenario has to be kept in mind when FE model results are interpreted. In our model, the weakest link is probably the uncertainty of exact muscle parameters for the cervid species. The next weakest link is the lack of specificity of the vocal tract shape in live animals.

Keeping these limitations in mind, high and low fundamental frequency calls were obtainable with the same virtual larynx, but with vastly different muscle activation and tissue strains (Figure 5). To obtain an elk bugle, a large stress had to be applied to stiffen the vibrating tissue. This required large cricothyroid muscle strength, as well as a robust vocal ligament to support stresses up to 10 MPa. For comparison, in normal human speech tissue stress of no more than about 400 kPa is required [54], but high pitch singing of a soprano singer also requires tissue stress in the MPa range by inference with Equation 5. To obtain a low fundamental frequency red deer call, cricothyroid activation needed to be low, but high lung pressure was needed to produce a loud utterance. A low frequency call is accompanied by large-amplitude vocal fold vibration and collision, both of which strained the tissue in shear as opposed to high tension in elk calls. High lung pressures can be assumed for the vocalizations in both cervid species.

At comparable lung pressures, the high frequency elk calls are acoustically most intense. The intensity of an acoustic signal is crucial for animal communication because a high signal-to-noise ratio improves signal reception and variation in amplitude may also have signaling value [e.g. 55], [56]. Considering that intensity is an important performance feature of vocal signals, the high fundamental frequency call could provide an efficiency advantage. An engineering-based approach of these very different vocal behaviors of elk and red deer allowed us to evaluate efficiency.

### The Efficiency of Producing High or Low Fundamental Frequency Calls

The energetic processes involved in producing a sound are complex [e.g. 57], [58], [59]. They include 1) the metabolic costs for generating respiratory driving pressures, 2) the metabolic costs for activating and contracting laryngeal muscles and 3) the metabolic costs for adjusting the upper vocal tract geometry. Requirements for respiratory metabolic energy are presumably similar between elk and red deer call production because very high lung pressures are necessary in both cases (Figure 7) and mating calls are uttered at high rates during the rut in both species [1], [2].

The efficiency of the conversion of aerodynamic to acoustic energy in the laryngeal sound source demonstrated differences. We found that the production of calls with high F_0_ seems to provide an efficiency advantage. Vocal efficiency in laryngeal sound production has been defined as the power radiated from the mouth divided by the aerodynamic power developed in the lungs [28], [60]. To produce a high amplitude call, the glottal area (air space between vocal folds) is much smaller in the high frequency call (Figure 7A) than in the low frequency call leading to less airflow through the glottis (Figure 7B). Consequently the aerodynamic power required is about two times smaller in the high frequency call (Figure 7C). Calculations for power radiated from the mouth suggest a 7 dB difference for a lung pressure of about 8 kPa (Figure 7D). The power radiated from the mouth is shown in Figure 1E. Taking the ratio of radiated power (Figure 7E) to aerodynamic power (Figure 7D) shows a five-fold efficiency advantage for the high frequency call (Figure 7F). This increased efficiency is based on two phenomena, a) the well-known more efficient radiation of higher frequencies from orifices (+6 dB/octave increase) [49], and b) a two-fold reduction in aerodynamic power required to drive *Cervid* vocal fold oscillation at high amplitudes. Part of the vocal fold tissue, the vocal ligament, is very stiff and prevents large glottal areas and glottal airflows at high fundamental frequencies.

The difference in glottal efficiency must be related to the metabolic energy to operate the sound source. The simulation indicates that the two species vocalize at two very distinct locations within a muscle activation plot. We might ask whether the different muscle activation result in different amounts of energy required to contract larynx muscles between the two species? Estimates (which remain speculative) suggest that there could be a difference in energy uptake for high versus low frequency sound production. A regular skeletal muscle demonstrates an ATP turnover rate of about 1 µmol ATP/g/sec during exercise [e.g. 61], [62], [63], [64]. 1 mol ATP provides about 29 kJ, which translates to about 0.03W/g. The TA muscle weighs about 4.9 g on each side (measured on the left and right side in a 5 year old male, left: 4.5 and right: 5.2 g) and the CT muscle is about 4.6 g on each side (measured on the left and right side in the same 5 year old male, left: 4.9 and right: 4.3 g). Both pairs of muscles are roughly 10 g each. If both muscles are functioning as regular skeletal muscles they would use about 0.3 W. The model suggests that for an elk call, the CT muscle must be activated to its maximum (at almost zero TA activity) while the red deer call requires approx. 50% TA activity (at zero CT activity) (Figure 5). Given that TA and CT muscles are comparable in size, and assuming that ATP turnover rate is proportional to muscle contraction intensity, this suggests a 2-fold difference in muscle energy uptake (“muscle metabolic rate”) giving the low F_0_ call a metabolic advantage (metabolic cost advantage of low F_0_ call 0.15W versus 0.3W). Even with an efficiency of muscles around 30% (muscle efficiency is the ratio of work and heat-plus-work, [65]), the metabolic advantage of producing low frequency calls is unlikely to be of a similar or greater magnitude as the aerodynamic power disadvantage (which for low F_0_ calls is 4W versus 2W, see Figure 7C at about 8kPa lung pressure). Furthermore, muscle efficiency is not indifferent to muscle fiber type. Fiber composition of TA, CT and other intrinsic laryngeal muscles are different [66] contributing to a more complex pattern of metabolic costs of force generation in the larynx.

### Signaling Fitness

Body size and muscular strength are important determinants of fitness in animals [67]. The utility of performance related measures (such as sound amplitude, fundamental frequency or formant frequency) lies in their link to fitness via selection. How does the low and high fundamental frequency dichotomy transfer to honest signaling? On the one hand, larger surfaces and longer tubes can resonate low-frequency sound effectively [68]. This principle seems to be exploited by the red deer to communicate size, and thereby fitness [5] and by many other species [e.g. 69], [70], [71], [72], [73]. On the other hand, radiation from surfaces and orifices is more efficient at high frequencies. Furthermore, the aerodynamic power required to achieve similar radiated sound amplitudes is lower in high frequency calls. An animal can obtain higher intensity by raising fundamental frequency. Some females respond to high intensity sounds as a signal of fitness [56], [74]. But to obtain a high F_0_ phonation with a large larynx, an enormous stress has to be applied to stiffen otherwise flaccid vibrating tissue. This requires extreme muscular strength, which listeners may interpret as the alternative fitness signal under the assumption that strength in the larynx relates to strength in the rest of the body. In humans, it is currently not clear which acoustic parameters in a persons' voice account for physical strength. Nevertheless perceptual experiments suggest that a human listener is able to assess a males' physical strength from just hearing the voice [75]. Thus, at least in humans, physical strength is predictable from the voice signal.

The vocal displays of other large mammals show also extreme vocal performances within and between species. For example, Chimpanzee (*Pan troglodytes*) panthoots are multi-call vocalizations, contrasting quieter and lower-pitched components with loud and high-pitched climax calls in the same bouts. As in our presented model for elk and red deer, a large F_0_ range can be produced with one and the same larynx design. Interestingly, highest ranking chimpanzee males are those who can drive the F_0_ of their voices to a maximum [76]. The vocal repertoire of the closely related Bonobo (*Pan paniscus*) demonstrates an even higher maximum F_0_ (e.g. [77]) paralleling the species dichotomy between elk and red deer.

Another example is our own species. Many utterances of human males cover a wide range of fundamental frequencies. Low human male voices are considered masculine and powerful, but voices in combat and romantic heroism (e.g. opera or heavy metal) are often high-pitched. Thus, for extreme vocal display (size or strength), it appears that F_0_ is driven to both extremes. However, when competition is not an issue (or is dealt with by means other than vocal combat), as in normal human conversation at close range, the middle of the voice range is accessed [78] probably because tissue deformation and respiratory effort is less costly.

The origin of the difference between elk and red deer male calling remains a fascinating, yet unresolved, phenomenon. A complex behavior such as vocal communication, is likely to have multiple constraints shaping it. The tradeoff between the advantage of high pitch sound production and the enormous laryngeal stress necessary seemingly works for the elk. Glottal efficiency increases with fundamental frequency in the Cervid larynx, thereby providing an advantage for high frequency vocalizations. Future studies also have to determine whether the auditory system of elk is better suited for high pitch than low pitch sounds. Other mechanisms, such as habitat acoustics or population density, along with the need to communicate over larger distances, must also be considered as driving forces.

## Supporting Information

Figure S1 Schematics of laryngeal cartilages from male elk. From each cartilage various measurements were taken. They are presented and further explained in Table S1. A and B: cricoid cartilage. C, D and E: thyroid cartilage. F,G, and H: arytenoid cartilage. The bar in the top left corner indicates a 1 cm distance.
(0.48 MB TIF)

Figure S2 Muscle activation plot (MAP) indicating 175 simulation results, each indicating the fundamental frequency.
(0.72 MB TIF)

Table S1 Summary of average data of laryngeal measurements from ten male elk and two red deer larynges. Values are means and standard deviations. Measurements on laryngeal cartilages are illustrated in Figure S1.
(0.05 MB DOC)

Text S1 Comparison of cartilage dimensions of elk and red deer.
(0.02 MB DOC)

## References

[1] Clutton-Brock TH, Albon SD (1979). The roaring of red deer and the evolution of honest advertising.. Behaviour.

[2] Struhsacker TT (1968). The behavior of the elk (*Cervus canadensis*) during the rut.. Z Tierpsychol.

[3] Bradbury JW, Vehrencamp SL (1998). Principles of Animal Communication..

[4] Schmidt-Nielsen K (1984). Scaling: Why is animal size so important?.

[5] Taylor AM, Reby D (2010). The contribution of source–filter theory to mammal vocal communication research.. J Zool.

[6] Alipour F, Titze IR (1999). Active and passive characteristics of the canine cricothyroid muscles.. J Voice.

[7] Story BH, Titze IR (1995). Voice simulation with a body cover model of the vocal fold.. J Acoust Soc Am.

[8] Riede T, Titze IR (2008). Vocal fold elasticity of the Rocky Mountain elk (*Cervus elaphus nelsoni*) – in the search for adaptations to produce high fundamental frequency vocalization with a large larynx.. J Exp Biol.

[9] Riede T, Lingle S, Hunter E, Titze IR (2010). Cervids with different vocal behavior demonstrate different visco-elastic properties of their vocal folds.. J Morph.

[10] Alipour F, Berry D, Titze IR (2000). A finite-element model of vocal-fold vibration.. J Acoust Soc Am.

[11] Cook DD, Mongeau L (2007). Sensitivity of a continuum vocal fold model to geometric parameters, constraints, and boundary conditions.. J Acoust Soc Am.

[12] Cook DD, Nauman E, Mongeau L (2008). Reducing the number of vocal fold mechanical tissue properties: Evaluation of the incompressibility and planar displacement assumptions.. J Acoust Soc Am.

[13] Cook DD, Nauman E, Mongeau L (2009). Ranking vocal fold parameters by their influence on model frequencies.. J Acoust Soc Am.

[14] Titze IR (2006). The Myoelastic-Aerodynamic Theory of Phonation.

[15] Titze IR, Strong W (1975). Normal modes in vocal cord tissue.. J Acoust Soc Am.

[16] Chan RW, Titze IR (1999). Viscoelastic shear properties of human vocal fold mucosa: Measurement methodology and empirical results.. J Acoust Soc Am.

[17] Hunter EJ, Titze IR (2007). Refinement in modeling the passive properties of laryngeal soft tissue.. J Appl Physiol.

[18] Gunter H (2003). A mechanical model of vocal-fold collision with high spatial and temporal resolution.. J Acoust Soc Am.

[19] Tao C, Jiang JJ, Zhang Y (2006). Simulation of vocal fold impact pressures with self-oscillating finite-element model.. J Acoust Soc Am.

[20] Hirano M (1974). Morphological structure of the vocal cord as a vibrator and its variations.. Folia Phoniatr (Basel).

[21] Hirano M (1975). Phonosurgery: Basic and clinical investigations..

[22] Kurita S, Nagata K, Hirano M, Bless DM, Abbs JH (1983). A comparative study of the layer structure of the vocal fold.. Vocal fold physiology..

[23] Titze IR, Jiang JJ, Lin E (1997). The dynamics of length change in canine vocal folds.. J Voice.

[24] Berry D, Reininger H, Alipour F, Bless DA, Ford CN (2005). Influence of vocal fold scarring on phonations: Predictions from a finite element model.. Ann Otol Rhinol Laryngol.

[25] Liljencrants S (1985). Speech synthesis with a reflection-type line analog..

[26] Titze IR, Story BH (2002). Rules for controlling low-dimensional vocal fold models with muscle activation.. J Acoust Soc Am.

[27] Titze IR (2008). Nonlinear source-filter coupling in phonation: Theory.. J Acoust Soc Am.

[28] Bouhuys A, Mead J, Proctor D, Stevens K (1968). Pressure-flow events during singing.. Annals of the New York Academy of Science.

[29] Fattu JM, Suthers RA (1981). Subglottic pressure and the control of phonation by the echolocating bat, Eptesicus.. J Comp Physiol.

[30] Rakesh V, Datta AK, Ducharme DG, Pease AP (2008). Simulation of Turbulent Airflow Using a CT Based Upper Airway Model of a Racehorse.. J Biomed Eng.

[31] Brown C, Alipour F, Berry DA, Montequin D (2003). Laryngeal biomechanics and vocal communication in the squirrel monkey (Saimiri boliviensis).. J Acoust Soc Am.

[32] Alipour F, Scherer RC, Finnegan E (1997). Pressure-flow relationships during phonation as a function of adduction.. J Voice.

[33] Alipour F, Jaiswal S (2008). Phonatory characteristics of excised pig, sheep, and cow larynges.. J Acoust Soc Am.

[34] Zhang Z, Neubauer J, Berry DA (2006). The influence of subglottal acoustics on laboratory models of phonation.. J Acoust Soc Am.

[35] Neder JA, Andreoni S, Lerario MC, Nery LE (1999). Reference values for lung function tests. II. Maximal respiratory pressures and voluntary ventilation.. Braz J Med Biol Res.

[36] Gömmel A, Butenweg C, Bolender K, Grunendahl A (2007). A muscle controlled finite-element model of laryngeal abduction and adduction.. Comput Methods Biomech Biomed Engin.

[37] Titze IR, Story BH (2002). Rules for controlling low-dimensional vocal fold models with muscle activation.. J Acoust Soc Am.

[38] Hunter EJ, Titze IR, Alipour F (2004). A three-dimensional model of vocal fold adduction/abduction.. J Acoust Soc Am.

[39] Titze IR, Hunter EJ (2007). A two-dimensional biomechanical model of vocal fold posturing.. J Acoust Soc Am.

[40] Köhler H (1984). Vergleichend-anatomische Untersuchungen am Kehlkopf von Cerviden..

[41] Story BH, Titze IR, Hoffman EA (1996). Vocal tract area functions from magnetic resonance imaging.. J Acoust Soc Am.

[42] Riede T, Tokuda IT, Munger JB, Thompson SL (2008). Mammalian laryngeal air sacs add variability to the vocal tract impedance: Physical and computational modeling.. J Acoust Soc Am.

[43] Story BH (1995). Speech simulation with an enhanced wave-reflection model of the vocal tract..

[44] Flanagan JL (1972). Speech Analysis, Synthesis and Perception. Second Edition.

[45] Titze IR (1988). The physics of small-amplitude oscillation of the vocal folds.. J Acoust Soc Am.

[46] Boersma P, Weenick D (2008). Praat: Doing phonetics by computer (Version 5.0.38) (Computer program).. http://www.praat.org.

[47] Berry DA, Herzel H, Titze IR, Krischer K (1994). Interpretation of biomechanical simulations of normal and chaotic vocal fold oscillations with empirical eigenfunctions.. J Acoust Soc Am.

[48] Titze IR (2000). Principles of Voice Production.

[49] Morse PM (1948). Vibration and Sound.

[50] Titze IR, Riede T, Popollo P (2008). Vocal exercises to determine nonlinear source-filter interaction.. J Acoust Soc Am.

[51] Zhang X, Jiang G, Wu C, Woo SL (2008). A subject-specific finite element model of the anterior cruciate ligament.. Conf Proc IEEE Eng Med Biol Soc.

[52] Kupczik K (2008). Virtual biomechanics: basic concepts and technical aspects of finite element analysis in vertebrate morphology.. J Anthropol Sci.

[53] Dumont ER, Grosse IR, Slater GJ (2009). Requirements for comparing the performance of finite element models of biological structures.. J Theor Biol.

[54] Titze IR, Luschei ES, Hirano M (1989). Role of the thyroarytenoid muscle in regulation of fundamental frequency.. J Voice.

[55] Gerhardt HC (1975). Sound pressure levels and radiation patterns of the vocalizations of some North American frogs and toads.. J Comp Physiol.

[56] Ritschard M, Riebel K, Brumm H (2010). Female zebra finches prefer high-amplitude song.. Anim Behav.

[57] Prestwich K (1994). The energetics of acoustic signaling in anurans and insects.. Am Zool.

[58] Oberweger K, Goller F (2001). The metabolic cost of birdsong production.. J Exp Biol.

[59] Gillooly JF, Ophir AG (2010). The energetic basis of acoustic communication.. Proc Roy Soc B.

[60] Blackbury JH (1977). Physiological energetics of cock-crow.. Nature.

[61] Katz A, Sahlin K, Henriksson J (1986). Muscle ATP turnover rate during isometric contraction in humans.. J Appl Physiol.

[62] Hochachka PW, Matheson GO (1992). Regulating ATP turnover rates over broad dynamic work ranges in skeletal muscles.. J Appl Physiol.

[63] Ratkevicius A, Quistorff B (2002). Metabolic costs of force generation for constant-frequency and catch-like-inducing electrical stimulation in human tibialis anterior muscle.. Muscle Nerve.

[64] Russ DW, Elliot MA, Vandenborne K, Walter GA, Binder-Macleod A (2002). Metabolic costs of isometric force generation and maintenance of human skeletal muscle.. Am J Physiol Endocrinol Metab.

[65] Smith NP, Barclay CJ, Loiselle DS (2005). The efficiency of muscle contraction.. Progress Biophys Mol Biol.

[66] Hoh JF (2005). Laryngeal muscle fibre types.. Acta Physiol Scand.

[67] Clutton-Brock TH, Clutton- Brock TH (1988). Reproductive success.. Reproductive success.

[68] Baranek LL (1954). Acoustics..

[69] Wallschläger D (1980). Correlation of song frequency and body weight in passerine birds.. Experientia.

[70] Gerhardt HC, Huber F (2002). Acoustic Communication in Insects and Frogs: Common Problems and Diverse Solutions.

[71] Hauser M (1994). The evolution of communication.

[72] Ryan M (1986). Factors influencing the evolution of acoustic communication: biological constraints.. Brain Behav Ecol.

[73] Fine ML, Thorson RF (2008). Use of passive acoustics for assessing behavioral interactions in individual toadfish.. Trans Amer Fish Soc.

[74] Ryan MJ (1988). Energy, calling and selection.. Amer Zool.

[75] Sell A, Bryant GA, Cosmides L, Tooby J, Sznycer D (2010). Adaptations in humans for assessing physical strength from the voice.. Proc Roy Soc B.

[76] Riede T, Arcadi AC, Owren MJ (2007). Nonlinear acoustics in pant hoots and screams of common chimpanzees (*Pan troglodytes*): Vocalizing at the edge.. J Acoust Soc Am.

[77] de Waal F (1988). The communicative repertoire of captive Bonobos (*Pan paniscus*), compared to that of Chimpanzees.. Behaviour.

[78] Hunter EJ, Svec JG, Titze IR (2006). Comparison of the produced and perceived voice range profiles in untrained and trained classical singers.. J Voice.

