# Supplementary figures and images for: A Cervid Vocal Fold Model Suggests Greater Glottal Efficiency in Calling at High Frequencies
Source: PLoS Comput Biol. 2010 Aug 19;6(8):e1000897. doi: 10.1371/journal.pcbi.1000897 (PMC2924247; doi:10.1371/journal.pcbi.1000897)

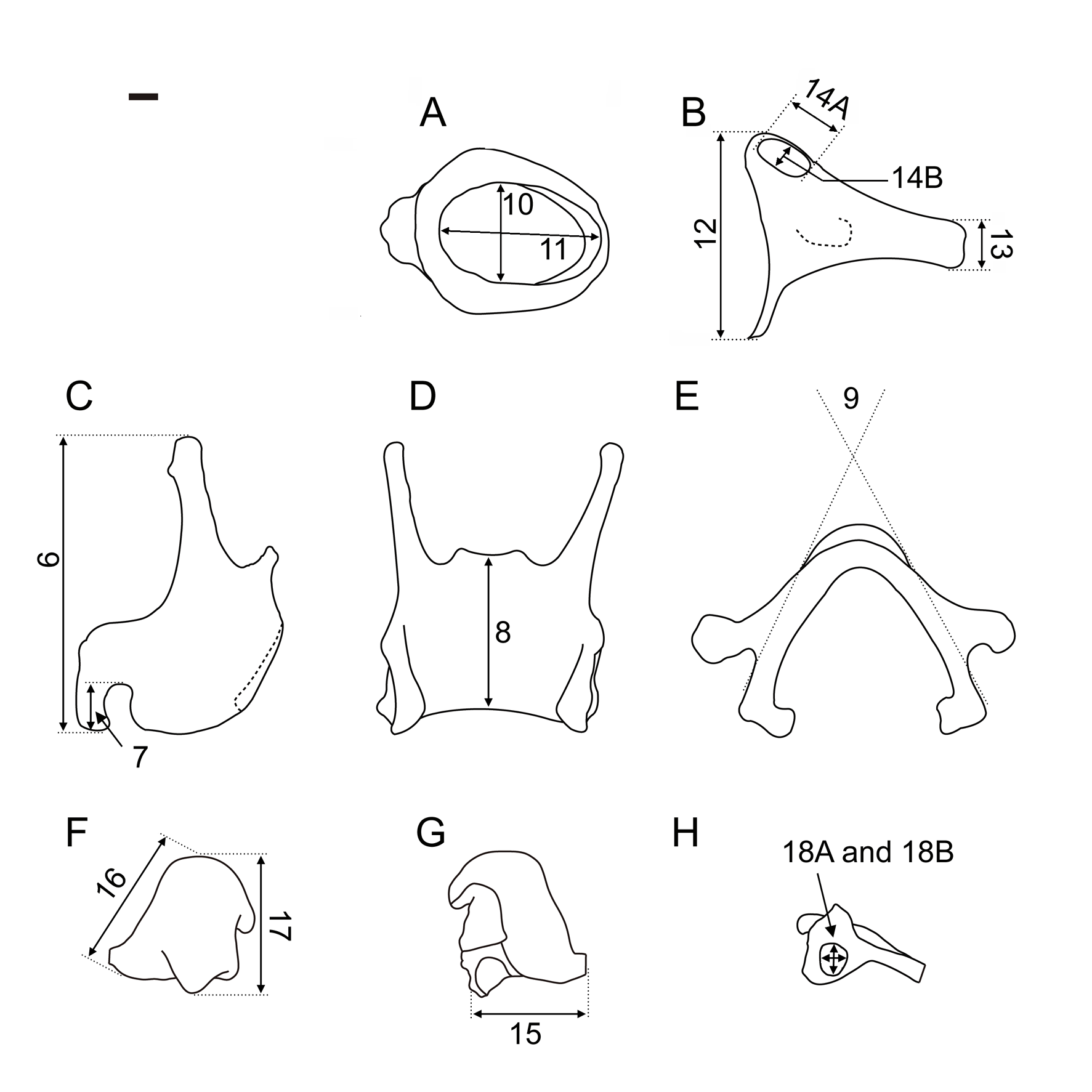

Supplement: Figure S1 — Schematics of laryngeal cartilages from male elk. From each cartilage various measurements were taken. They are presented and further explained in Table S1. A and B: cricoid cartilage. C, D and E: thyroid cartilage. F,G, and H: arytenoid cartilage. The bar in the top left corner indicates a 1 cm distance. (0.48 MB TIF) [file pcbi.1000897.s001.tif]

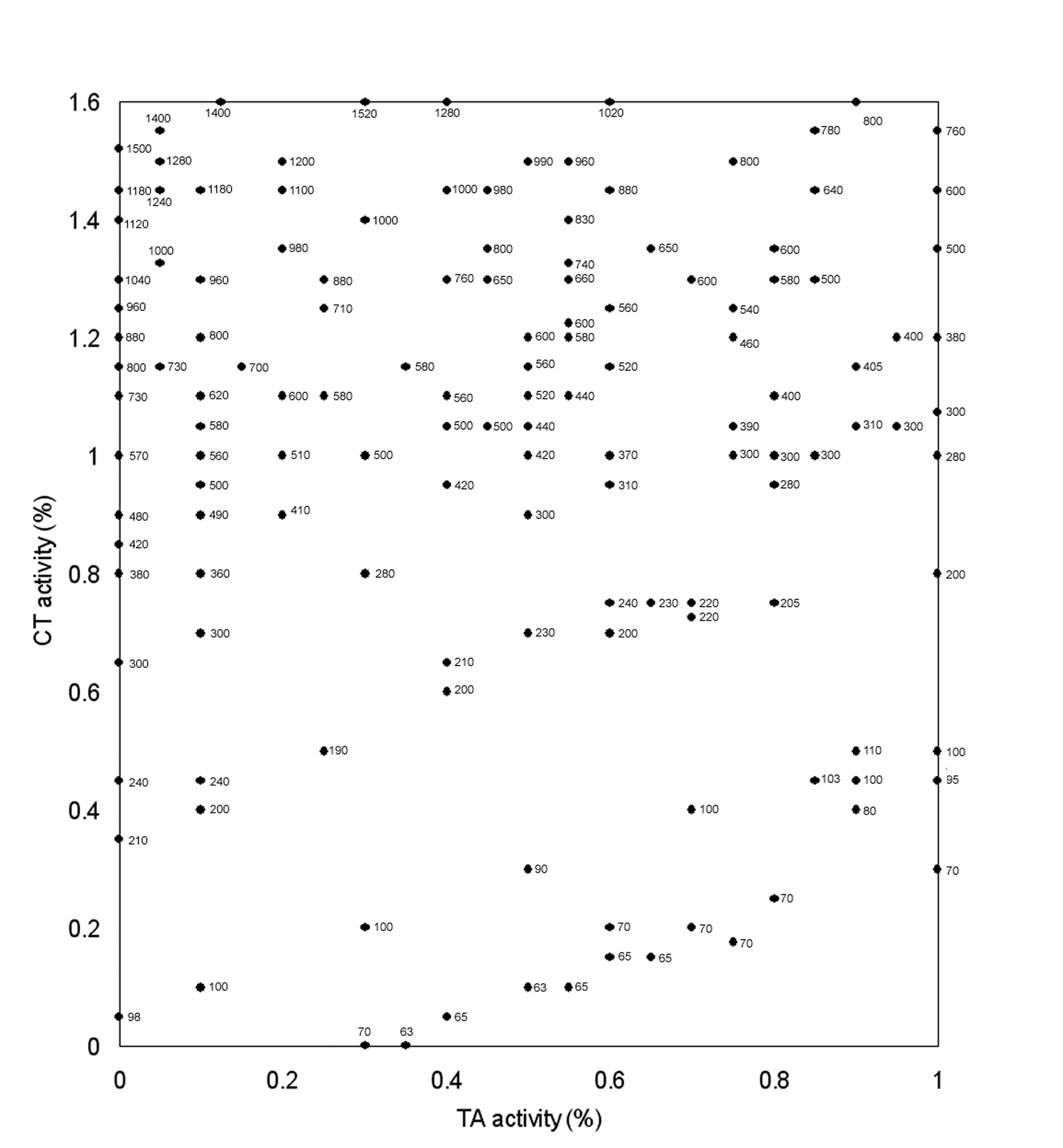

Supplement: Figure S2 — Muscle activation plot (MAP) indicating 175 simulation results, each indicating the fundamental frequency. (0.72 MB TIF) [file pcbi.1000897.s002.tif]
