# Supplementary material for: A Cervid Vocal Fold Model Suggests Greater Glottal Efficiency in Calling at High Frequencies
Source: PLoS Comput Biol. 2010 Aug 19;6(8):e1000897. doi: 10.1371/journal.pcbi.1000897 (PMC2924247; doi:10.1371/journal.pcbi.1000897)
Supplement: Table S1 — Summary of average data of laryngeal measurements from ten male elk and two red deer larynges. Values are means and standard deviations. Measurements on laryngeal cartilages are illustrated in Figure S1. (0.05 MB DOC) [file pcbi.1000897.s003.doc]

Table S1: Summary of average data of laryngeal measurements from ten male elk and two red deer larynges. Values are means and standard deviations. Measurements on laryngeal cartilages are illustrated in Figure S1.

|  | **Description of parameters** | **elk** | **red deer** |
| --- | --- | --- | --- |
| 1 | Age (years) | 2.9±0.7 | 4.0±1.4 |
| 2 | Vocal fold length (cm) | 3.0±0.3 | 2.7±0.1 |
| 3 | Weight thyroid cartilage (g) | 40.1±5.9 | 35.5±0.7 |
| 4 | Weight cricoid cartilage (g) | 33.9±4.8 | 29.3±0.3 |
| 5 | Weight arytenoid cartilage (g) | 14.8±2.0 | 12.5±0.7 |
|  | **Thyroid cartilage** |  |  |
| 6 | Distance between tips of cranial and caudal cornua (cm) | 10.5±0.5 | 9.3±0.3 |
| 7 | Distance between tip of caudal cornu and base of cornu (cm) | 1.7±0.2 | 1.6±0.7 |
| 8 | Distance between caudal and cranial edge of ventral commisure (cm) | 5.3±0.3 | 5.0±0.1 |
| 9 | Angle of thyroid ala in dorsal view (º) | 67.2±4.9 | 64.0±2.8 |
|  | **Cricoid cartilage** |  |  |
| 10 | Latero-lateral inner diameter (cm) | 3.5±0.3 | 3.3±0.1 |
| 11 | Dorso-ventral inner diameter (cm) | 5.3±0.3 | 5.0±0 |
| 12 | Dorsal height (cm) | 6.5±0.5 | 5.9±0.1 |
| 13 | Ventral height (cm) | 1.2±0.1 | 1.1±0.1 |
| 14A | Latero-lateral dimension of the crico-arytenoid joint (cm) | 1.9±0.2 | 1.7±0.03 |
| 14B | Dorso-ventral dimension of the crico-arytenoid joint (cm) | 0.9±0.1 | 0.8±0.01 |
|  | **Arytenoid cartilage** |  |  |
| 15 | Vocal process to muscular process (cm) | 3.4±0.2 | 3.3±0.1 |
| 16 | Vocal process to Apex (cm) | 5.1±0.3 | 5.0±0.1 |
| 17 | muscular process to Apex | 4.1±0.3 | 3.9±0.1 |
| 18A | Latero-lateral dimension of the crico-arytenoid joint facet dimension (cm) | 1.3±0.1 | 1.2±0.1 |
| 18B | Dorso-ventral dimension of the crico-arytenoid joint facet dimension (cm) | 0.9±0.1 | 0.9±0.03 |
