# Supplementary material for: A Cervid Vocal Fold Model Suggests Greater Glottal Efficiency in Calling at High Frequencies
Source: PLoS Comput Biol. 2010 Aug 19;6(8):e1000897. doi: 10.1371/journal.pcbi.1000897 (PMC2924247; doi:10.1371/journal.pcbi.1000897)
Supplement: Text S1 — Comparison of cartilage dimensions of elk and red deer. (0.02 MB DOC) [file pcbi.1000897.s004.doc]

**Text S1**

Comparison of cartilage dimensions of elk and red deer

With a caliper (+/- 0.1 mm accuracy), the length of the vocal fold was measured from the insertion of the vocal fold at the thyroid cartilage to the tip of the vocal process of the arytenoid cartilage. Note that the in situ vocal fold length is measured in the intact larynx (not cut open). Access to the vocal fold for this in situ measurement is possible by cutting a window (2 mm x 5 mm), mid-sagital at the cranio-dorsal edge of the cricoid cartilage. This allows access for a small narrow caliper, paralleling the vocal fold in its complete length, without the need to stretch or bend any structure of the larynx framework. The length epilarngeal tube was measured from the cranial edge of the vocal fold to the cranial edge of the plica aryepiglottica.

After vocal fold and epilarynx tube length were taken, laryngeal cartilages (epiglottis, thyroid, 2 arytenoid, cricoid) were freed from soft tissue, weighted and a collection of 15 linear parameters were measured with a caliper (+/- 0.1 mm accuracy). The angle of the thyroid cartilage was also measured. The parameters taken are further explained in Figure S1 and presented in Table S1.
